# Retrospective multicentre evaluation of common calcaneal tendon injuries in 66 cats. Part 1: study population, injury specification and classification

**DOI:** 10.1177/1098612X221131253

**Published:** 2023-01-27

**Authors:** Thomas C Häußler, Matthias Kornmayer, Miriam Scheich, Andreas Fischer, Christian J Feichtenschlager, Thomas Rohwedder

**Affiliations:** 1 Department of Veterinary Clinical Sciences, Small Animal Clinic – Surgery, Justus-Liebig-University, Giessen, Germany; 2 Clinic for Small Animal Surgery and Reproduction, Ludwig-Maximilians-University, Munich, Germany; 3 Small Animal Clinic Hofheim, Hofheim, Germany; 4 Small Animal Clinic Kalbach, Kalbach, Germany; 5 Small Animal Clinic, Free University Berlin, Berlin, Germany

**Keywords:** Common calcaneal tendon, Achilles tendon, tendinopathy, rupture

## Abstract

**Objectives:**

The objective of the first part of this retrospective multicentre study was to identify and classify common calcaneal tendon (CCT) injuries in a study population of 66 cats.

**Methods:**

The medical records of five different small animal referral centres and veterinary teaching hospitals between 2010 and 2020 were reviewed. In addition to patient-specific data, CCT injuries were characterised in detail. Diagnostic modalities and further comorbidities were recorded.

**Results:**

Sixty-six cats met the inclusion criteria. The mean age of the cats was 7.5 years (range 0.5–16.3) and their mean body weight (BW) was 4.6 kg (range 1.5–9.0). Thirty-four spayed females (51.5%), five intact females (7.6%) and 27 castrated males (40.9%) were included. Most cases involved closed injuries of the CCT (69.7%). Twenty-one of 46 cats had closed atraumatic injuries (45.7%). Open injuries (30.3%) were most commonly lacerations (65%). Twenty-one injuries were classified as atraumatic (31.8%), whereas 25 were traumatic (37.9%). With every year of age, the odds of having an atraumatic injury increased by a factor of 1.021. Cats with atraumatic injuries had a higher mean BW than cats with traumatic injuries, but the difference was not statistically significant. Acute injuries were recorded in 40.9% of cases, whereas 51.5% of cats had a subacute CCT injury and 7.6% had chronic lesions. Most acute lesions were Meutstege type I injuries (55.6%). Subacute and chronic lesions were more commonly Meutstege type IIc injuries (58.8% and 60%, respectively). Considering all CCT injuries, a Meutstege type IIc injury was most common (53%).

**Conclusions and relevance:**

The most common type of injury was Meutstege type IIc. Cats with atraumatic injuries had a higher mean BW than cats with traumatic injuries, but the difference was not statistically significant. Older cats more commonly presented with atraumatic CCT injuries.

## Introduction

Injuries to the common calcaneal tendon (CCT), also known as the Achilles tendon (AT), are considered a rare condition in feline orthopaedics.^1–3^ Very few reports exist about treatments and outcomes, and those available include only a small number of cases, making it difficult to draw conclusions. Meutstege published a classification system of CCT injuries in dogs,^4^ which is also used in cats.^3^ Type I lesions involve a complete tendon tear, including all parts of the CCT. Type IIa lesions are musculotendinous ruptures, type IIb lesions include ruptures of the AT with intact paratenon and type IIc lesions define gastrocnemius tendon (GT) avulsions with an intact superficial digital flexor tendon (SDFT). Type III lesions include tendinosis or peritendinitis.^4^ An alternative time-dependent classification system was proposed by Reinke et al.^5^ In this classification system, an acute injury is defined as one in which there is <2 days between the injury and surgery. Subacute lesions are characterised by 2–21 days between injury and surgery, and chronic lesions by >21 days between injury and surgical treatment.

In cats, most ruptures are due to closed avulsion injuries from the tuber calcanei. CCT injuries lead to non-weightbearing lameness and hyperflexion of the tarsal joint.^1–3,6,7^ With the stifle joint in extension, the hock can be fully flexed in cases of complete rupture of the CCT, whereas partial ruptures display less tarsal hyperflexion. If the SDFT remains intact, the phalangeal joints of the hindlimb will flex simultaneously, resulting in the typical ‘claw-like’ appearance while standing. Other clinical findings include thickening and loss of continuity of the tendon structures, as well as external wounds in traumatic cases.^2,4,8^ In most cases, surgical repair of the tendon and temporary tibiotarsal joint immobilisation are recommended, and several techniques have been described.^2,6–13^ Only one report has described AT injuries, surgical technique and immobilisation, as well as short- and long-term outcomes in cats.^3^ In that report, geriatric female cats seemed to be predisposed to atraumatic tendinosseous avulsions. Obesity could not be determined as a causative factor in AT pathogenesis, and the conclusions were statistically limited owing to the small number of cases (21 cats).^3^ Therefore, the aim of the present study was to collect a considerably larger sample size and to compare clinical data, examination findings, injury specifications and injury classifications. We hypothesised that older and obese cats are more prone to atraumatic AT injuries.

## Materials and methods

### Data acquisition

This retrospective multicentre study included the medical records of five different small animal clinics in Germany (Free University of Berlin; Ludwig Maximilians University Munich; Justus Liebig University Giessen; Small Animal Clinic Hofheim; Small Animal Clinic Kalbach) between 2010 and 2020. The obtained data (see Table 1 in the supplementary material) comprised signalment, body weight (BW), medical history and orthopaedic examination findings, including the type of injury and trauma. Open and closed injuries were differentiated, and trauma was further characterised as laceration, biting injuries, high-rise trauma, trauma that occurred during running/being chased, blunt major injuries (car accident), blunt minor injuries (eg, a crushing injury if the limb was caught in a door or window frame) and atraumatic injuries. Atraumatic injuries were defined as those without any wound (closed injury) and occurring during normal physiological activity such as running or jumping from indoor objects (eg, furniture, a scratching tree) or without any evidence of trauma in indoor cats. All open and closed injuries of a high-energy trauma origin (high-rise trauma, vehicular trauma) were categorised as traumatic. Time to surgery, lameness grade, stance type and other clinical findings, as well as further diagnostic modalities to achieve a diagnosis of AT injury, were documented. Comorbidities such as obesity, cardiomyopathies, endocrinopathies or neoplastic diseases were documented if the information was available.

### Statistical analysis

The mean ± SD values for age, BW at time of surgery and time between injury and surgery were calculated and evaluated. Age, BW and sex were evaluated for their influence on injury type, but only age and BW remained in the final model. Five intact cats were excluded for the evaluation of the variable sex, because they represented a low proportion of the total population.

Stepwise backward elimination was carried out to build the model. The first model included all possible independent variables. Next, the independent variable with the highest *P* value was excluded and the model was rerun. This step was repeated until the best-fitting model with the smallest Akaike information criterion, Schwarz criterion and negative two times the log-likelihood was found. A *P* value <0.05 was considered to be statistically significant, and a *P* value <0.001 was considered to be highly statistically significant.

## Results

### Study population

In total, 70 cats with common calcaneal tendinopathies were found in the medical records. After the exclusion of four Maine Coons due to increased body size and increased BW without being obese, 66 cats were included in the final evaluation (59 domestic shorthair cats [89.4%]; see Table 1 in the supplementary material). At the time of data collection, the mean ± SD age was 7.5 ± 4.7 years (range 0.5–16.3) and patients had a mean ± SD BW of 4.6 ± 1.2 kg (range 1.5–9.0). Most cats were female (59.1%) (Table 1).

**Table 1:** Signalment and trauma in obese vs non-obese cats in the total study population

	Obese cats (n = 26 [39.4%])	Non-obese cats (n = 40 [60.6%])	Total (n = 66)
BW (kg)	5.7 ± 1.0 (4.6–9)	3.9 ± 0.7 (1.5–4.8)	4.6 ± 1.2 (1.5–9)
Age (years)	9.1 ± 3.8 (1.5–15.3)	6.5 ± 5.0 (0.5–16.3)	7.5 ± 4.7 (0.5–16.3)
Indoor cats	13 (50)	15 (37.5)	28 (42.4)
Outdoor cats	13 (50)	25 (62.5)	38 (57.6)
Sex			
FI	1 (3.8)	4 (10)	5 (7.6)
FS	11 (42.3)	23 (57.5)	34 (51.5)
MC	14 (53.8)	13 (32.5)	27 (40.9)
Injury classification			
Open	8 (30.8)	12 (30)	20 (30.3)
Closed	18 (69.2)	28 (70)	46 (69.7)
Traumatic	8 (30.8)	17 (42.5)	25 (37.9)
Atraumatic	11 (42.3)	10 (25)	21 (31.8)
Not defined as traumatic/atraumatic	7 (26.9)	13 (32.5)	20 (30.3)
Meutstege classification of the atraumatic group			
I	–	2 (20)	2 (9.5)
IIa	1 (9.1)	–	1 (4.8)
IIb	1 (9.1)	–	1 (4.8)
IIc	8 (72.7)	7 (70)	15 (71.4)
III	–	1 (10)	1 (4.8)
Not defined	1 (9.1)	–	1 (4.8)
Meutstege classification of the traumatic group			
I	7 (87.5)	11 (64.7)	18 (72)
IIa	–	–	–
IIb	–	–	–
IIc	1 (12.5)	6 (35.3)	7 (28)
III	–	–	–
Not defined	–	–	–

Data are presented as n (%) or mean ± SD (range)

BW = body weight; FI = female intact; FS = female spayed; MC = male castrated

### Examination

All cats underwent a complete clinical and orthopaedic examination. Further diagnostic work-up to evaluate the CCT injury varied among the patients. Twenty (30.3%) cats underwent surgery immediately without any further diagnostic imaging. In those cases, the extent of the injury was evaluated intraoperatively. Forty-six (69.7%) cats had preoperative radiographs showing the whole lower limb and 22 (33.3%) cats had additional preoperative ultrasonographic evaluation of the AT.

### Injury specifications

Most cases presented with a closed injury of the CCT (n = 46 [69.7%]). Twenty-five (37.9%) cats showed a closed injury of unknown origin. In 21 (31.8%) cats, closed injuries: were atraumatic, resulting from jumping off the sofa or a cat stand (n = 14 [21.2%]); resulted from running or being chased (n = 2 [3.0%]); or were due to blunt minor injuries, such as catching or crushing injuries (n = 3 [4.5%]). Twenty (30.3%) cats presented with an open injury such as laceration injuries (n = 13 [19.7%]). Other types of trauma were less common (Table 2).

**Table 2:** Clinical examination findings and classification of injury type in 66 cats with common calcaneal tendinopathy

Laterality	
Right limb	32 (48.5)
Left limb	34 (51.5)
Injury type	
Closed injury	46 (69.7)
Unknown origin	25 (54.3)
Jumping off furniture/cat stand	14 (30.4)
Catching/crushing injury	3 (6.5)
Running/being chased	2 (4.3)
High-rise trauma	2 (4.3)
Open injury	20 (30.3)
Unknown origin	3 (15)
Laceration/cut injury	13 (65)
High-rise trauma	2 (10)
Biting injury	1 (5)
Car accident	1 (5)
Stance	
Moderate-to-full plantigrade stance with bunching of the digits	42 (63.6)
Full plantigrade stance	24 (36.4)
Lameness grade (0–4 scale)^14^	
1	–
2	39 (59.1)
3	13 (19.7)
4	14 (21.2)
Other clinical findings	
Swelling at the level of CCT injury	51 (77.3)
Palpable gap	47 (71.2)
Pain	36 (54.6)
Palpable bony fragment proximal to the calcaneus	3 (4.5)
Combination	47 (71.2)

Data are presented as n (%)

CCT = common calcaneal tendon

A 0–4-scale lameness grading system was used.^15^ Most cats presented with grade 2/4 lameness (n = 39 [59.1%]). Only 14 cats showed a permanent non-weightbearing lameness grade 4/4 (21.2%; Table 2). The distribution of injuries according to the classification by Meutstege^4^ is shown in Table 3. Twenty-one injuries (31.8%) were classified as atraumatic, whereas 25 (37.9%) were traumatic. Twenty (30.3%) could not be classified clearly, including cats with a closed injury but outdoor access and therefore lacking clear determination or exclusion of any traumatic event. One cat with GT avulsion (type IIc) showed an intact SDFT but with lateral luxation. Information on the BW, age and sex distribution of cats in these groups is provided in Table 4.

**Table 3:** Distribution of the type of tendon injury according to the Meutstege classification^4^ in the atraumatic and traumatic groups and the total study population*

Meutstege Classification	Type of injury		
	Atraumatic (n = 21)	Traumatic (n = 25)	Total (n = 66)
I	2 (9.5)	18 (72)	25 (37.9)
IIa	1 (4.8)	–	3 (4.5)
IIb	1 (4.8)	–	1 (1.5)
IIc	15 (71.4)	7 (28)	35 (53)
III	1 (4.8)	–	1 (1.5)
Not defined	1 (4.8)	–	1 (1.5)

Data are presented as n (%)

* Twenty cases could not clearly be classified as atraumatic or traumatic in origin

**Table 4:** Body weight (BW), age and sex distribution of cats in the atraumatic and traumatic groups, and total study population

	Type of injury			
	Atraumatic (n = 21 [31.8%])	Traumatic (n = 25 [37.9%])	Not classified (n = 20 [30.3%])	Total (n = 66)
BW (kg)	5.2 ± 1.5 (3.0–9.0)	4.3 ± 1.1 (1.5–6.4)	4.4 ± 0.7 (3–5.3)	4.6 ± 1.2 (1.5–9.0)
Age (years)	10.4 ± 4.0 (0.5–15.3)	5.2 ± 4.3 (0.6–15.8)	7.5 ± 4.2 (0.5–16.3)	7.5 ± 4.7 (0.5–16.3)
Sex				
FI	1 (4.8)	2 (8)	2 (10)	5 (7.6)
FS	10 (47.6)	13 (52)	11 (55)	34 (51.5)
MC	10 (47.6)	10 (40)	7 (35)	27 (40.9)

Data are presented as n (%) or mean ± SD (range)

FI = female intact; FS = female spayed; MC = male castrated

During the entire study period, only three cats became bilaterally affected. These cats were only included for a single limb because the later contralateral injury was treated elsewhere.

In the logistic regression model there was a statistically significant influence of age on the occurrence of traumatic vs atraumatic injuries (*P* = 0.006). With every year of age, the odds of having an atraumatic injury as opposed to a traumatic injury increased by a factor of 1.021. The association between age and BW was evaluated by using Spearman’s rank correlation coefficient. There was a statistically significant correlation between the variables age and BW in the traumatic injury group (*r* = 0.47, *P* = 0.018) but not in the atraumatic injury group, allowing for the conclusion that in the traumatic injury group younger cats had a significantly lower mean BW compared with older cats.

### Time-dependent injury classification

When injuries were time-dependently classified according to Reinke et al,^5^ 27 cats (40.9%) sustained an acute AT injury, 34 (51.5%) were categorised as having a subacute CCT injury and five (7.6%) presented with a chronic lesion. Twenty-eight of the 34 subacute injuries (82.4%) were closed, with 16 (57.1%) of unknown origin and eight (28.6%) being atraumatic (due to jumping off furniture or a cat stand). Four of the five chronic injuries (80%) were closed, with three (75%) being atraumatic in origin. Cats that presented with an acute condition usually had higher grades of lameness at the time of presentation than chronic cases. All acute injuries showed other clinical findings such as swelling, pain or a palpable gap at the lesion site. Fifteen acute injuries (55.6%) were Meutstege type I lesions, 20 subacute injuries (58.8%) were Meutstege type IIc lesions and three chronic injuries (60.0%) were Meutstege type IIc lesions (Table 5).

**Table 5:** Time-dependent injury classification in 66 cats, according to Reinke et al^5^

	Acute injures (n = 27)	Subacute injuries (n = 34)	Chronic injuries (n = 5)
Closed	14 (51.9)	28 (82.4)	4 (80)
Unknown origin	8 (57.1)	16 (57.1)	1 (25)
Jumping off furniture/cat stand	3 (21.4)	8 (28.6)	3 (75)
Catching/crushing injury	1 (7.1)	2 (7.1)	–
Running/being chased	1 (7.1)	1 (3.6)	–
High-rise trauma	1 (7.1)	1 (3.6)	–
Open	13 (48.1)	6 (17.6)	1 (20)
Unknown origin	1 (7.7)	1 (16.7)	1 (100)
Laceration injury	10 (76.9)	3 (50)	–
High-rise trauma	1 (7.7)	1 (16.7)	–
Biting injury	–	1 (16.7)	–
Car accident	1 (7.7)	–	–
Lameness grade: 0–4 scale^14^			
1	–	–	–
2	12 (44.4)	22 (64.7)	5 (100)
3	4 (14.8)	9 (26.5)	–
4	11 (40.7)	3 (8.8)	–
Other clinical findings			
Swelling at the level of CCT injury	27 (100)	23 (67.7)	1 (20)
Palpable gap	24 (88.9)	21 (61.8)	2 (40)
Pain	23 (85.2)	13 (38.2)	1 (20)
Palpable bony fragment proximal to the calcaneus	1 (3.7)	2 (5.9)	–
Combination	26 (96.3)	21 (61.8)	–
Meutstege classification			
Type I	15 (55.6)	11 (32.4)	–
Type IIa	1 (3.7)	1 (2.9)	1 (20)
Type IIb	–	–	1 (20)
Type IIc	11 (40.7)	20 (58.8)	3 (60)
Type III	–	1 (2.9)	–

Data are presented as n (%)

CCT = common calcaneal tendon

### Obesity

Twenty-six of 66 (39.4%) cats were categorised as obese in their medical records. Body condition score (BCS) was not determined in most cases. The cats had a mean BW of 5.7 ± 1.0 kg (range 4.6–9) and a mean age of 9.1 ± 3.8 years (range 1.5–15.3). All traumatic injuries (n = 8 [30.8%]) were open injuries due to laceration or high-rise trauma. Seven closed injuries (26.9%) could not be classified more precisely, because the accidents were not observed by the owners and all cats were outdoor cats. All other patients in the obese group (n = 11 [42.3%]) had atraumatic injuries. The most common injury type in this group was Meutstege type IIc (n = 8 [72.7%]). Signalment and trauma details were compared with those of non-obese cats, as well as the total population and are presented in Table 1. Neither BW nor obesity had a significant influence on the occurrence of traumatic vs atraumatic injuries.

## Discussion

CCT injuries are reported to be uncommon in cats,^3^ with only a few cases published in the veterinary literature.^1,3,6,7,16^ On the basis of our retrospective multicentre evaluation, we can confirm that CCT injuries remain a rare orthopaedic condition in cats.

**Figure 1:**
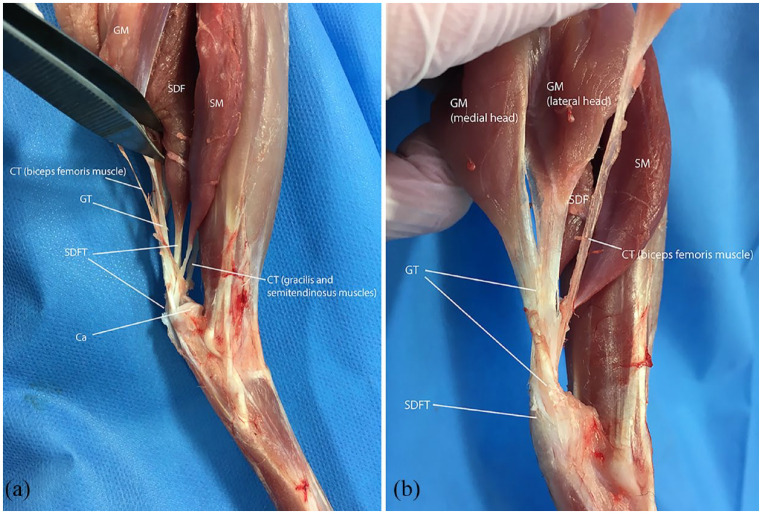
(a) Lateral view of the dissected feline crus with focus on the common calcaneal tendon and its insertion onto the calcaneus (Ca). Up is proximal, down is distal, right is cranial and left is caudal. (b) Caudolateral close-up view of the common calcaneal tendon (CCT) proximal to the level of the calcaneus. The superficial digital flexor tendon (SDFT) widens in superficial position forming a cap and attaches on either side on the tuber calcanei. The gastrocnemius muscle (GM) with its medial and lateral head forms the gastrocnemius tendon (GT). The superficial digital flexor muscle (SDF) is directly cranial to the GM. The soleus muscle (SM) is the most cranial muscle participating the CCT. The combined tendon of the biceps femoris muscle (lateral) and the gracilis and semitendinosus muscles (medial; CT) portion of the biceps femoris muscle runs from lateral via cranial the other components of the CCT to its medial attachment site of the calcaneus where it converges with the CT portion of the gracilis and semitendinosus muscles

The Meutstege classification system^4^ does not fully account for cats because it disregards their specific anatomy. Cats exhibit an additional component of the CCT, namely the soleus muscle (Figure 1). Published data on CCT injuries in cats lack detailed information about the affected parts after injury.^3,6,7,16^ Unfortunately, the same was true in the present study, as the medical records only contained Meutstege types without any consideration of the soleus muscle tendon (SMT). Future studies should concentrate on collecting detailed information on ruptured tendon parts and the clinical consequences of various combinations of rupture types.

In humans, CCT injuries are typically observed with a male to female ratio from 2:1 to 12:1.^17^ In contrast, Cervi et al^3^ reported that 91% of their feline study population was female. Our study population was more balanced (59.1% female, 40.9% male). Most of the cats were spayed or castrated; consequently, the impact of neutering could not be evaluated in our study. In humans, as many as 84% of all AT ruptures occur in men.^18^ A rodent model showed that this can be explained by inferior mechanical properties of the AT and increased muscle fibre size in men vs women. Increased muscle fibre size means greater muscle strength and the combination could be responsible for the increased rate of spontaneous tendon ruptures in male patients.^19^ Nevertheless, female cats were, again, over-represented in the present study. Gonadectomy increases the incidence of musculoskeletal disorders in dogs.^14,20^ There is an increased risk for cranial cruciate ligament tears in for gonadectomised dogs,^20^ and activated luteinising hormone receptors in the ligament may be responsible for the higher rate of tears in neutered dogs.^21^ This is also a possible explanation for the high rate of AT injuries in spayed female cats. In human orthopaedics, commonly reported mechanisms for AT rupture are rapid extension of the tarsus during weightbearing with extension of the stifle joint, dorsiflexion of the ankle, and rapid or violent dorsiflexion of the tarsal joint,^22,23^ as well as precursory inflammatory or non-inflammatory tendinosis with collagen fibre degeneration.^24^

In humans, blood flow decreases with age at the most susceptible region of the AT, namely 3–6 cm proximal to the calcaneal insertion. Further, the tensile strength of collagen decreases over time.^23^ The ability to withstand repetitive stress forces decreases with increasing tissue stiffness during ageing, and less strain is required to cause collagen failure.^22^ Therefore, levels of activity must be adapted to the level of fitness of the individual. A more sedentary lifestyle leading to degenerative changes in the AT, such as mucoid and hypoxic degeneration, calcifications, tendolipomatosis, degenerated collagen fibres and tissue necrosis, have been discussed as possible causative factors in human sports medicine.^25,26^ This fact may explain the high occurrence rates of atraumatic injuries in indoor cats, assuming they are less active than their outdoor counterparts. Being less adapted to physical exertion, minor activities such as jumping or running indoors may lead to mechanical overload of the tendon more quickly.Obesity is another cause that may lead to musculotendinous overload during locomotion, ultimately resulting in tendon rupture.^27^ In our study, 39.4% of cats were categorised as obese. These cats had a mean BW of 5.7 kg. For comparison, Cervi et al^3^ reported a mean BW of 5.4 kg for cats with atraumatic lesions. In our study, cats with atraumatic lesions had a mean BW of 5.2 kg. Evaluation of the BCS would have been more precise than the subjective documentation of being obese. Unfortunately, the BCS was not reliably recorded. We had no control group comprising obese cats that did not develop AT injuries, so we can only assume that increased BW may play a role in feline AT rupture pathogenesis. In contrast to Cervi et al,^3^ closed injuries in outdoor cats, where a traumatic event could not be ruled out, were excluded from further analysis, and not automatically counted as atraumatic. Interestingly, most atraumatic injuries were type IIc lesions (71.4%) with an avulsed GT and intact SDFT, a finding that corresponds to the results reported by Cervi et al,^3^ namely 66.7% tendinosseous avulsions. Only 9.5% were complete ruptures of the CCT (Table 3).

There was no clear sex predisposition for atraumatic lesions, with 52.4% being female (Table 4). In the logistic regression model, age had a statistically significant influence on the type of injury. With increasing age the occurrence of atraumatic injuries increased. There was a significant Spearman’s rank correlation between age and BW (*r* = 0.47, *P* = 0.018); however, the relationship was not strong owing to a high level of scatter in the scatter diagram, indicating a lesser degree of correlation between variables. In the traumatic group, younger cats tended to have a lower BW than older ones. This relationship could not be found for the atraumatic group and had no influence on statistical analysis of the variable BW for both groups. Based on our analysis, we could not identify increased BW or obesity as causative factors for AT pathologies in cats. With more cases and better evaluation of BCS, the informative power would have been greater. Molecular studies regarding possible reasons for chronic tendon degeneration are not yet available for feline or canine patients. Human studies are promising, showing specific expression patterns of protease-activated receptors (PARs) and their general role together with mast cell activation in tendinopathies.^28^ Future veterinary studies may focus on PARs as a possible target for CCT treatment.

For the time-dependent classification of the AT injuries, we referred to Reinke et al.^5^ Many acute injuries were open, which explains the early presentation to the clinic. There was a high number of subacute presentations (51.5%); most were Meutstege type IIc lesions (58.8%) and 85.7% were categorised as atraumatic or due to trauma of unknown origin. With type IIc lesions, cats are still able to ambulate owing to an intact SDFT. That may explain the delayed presentation of those cases because the owners did not recognise trauma and considered the lameness to be ‘not severe’. It is not known whether the SMT remains intact in cats with type IIc lesions. Future studies should focus on SMT status in different types of AT injury. Of note, 48.1% of acute injuries, 17.6% of subacute injuries and 20% of chronic injuries were open (Table 5). The high proportion of open injuries in the acute category may be explained by the assumption that owners who recognise a wound might present the cat earlier to a veterinarian, compared with a cat only showing lameness or a gait abnormality.

There are multiple limitations to our study. The study was retrospective in nature. Although it was a multicentre study – to increase the number of patients – the number of patients was still too small to make strong conclusions about predisposing factors such as sex and BW. Considering that we evaluated five clinics over a 10-year period and still did not have enough patients for evaluation, future studies should encompass even more clinics or a longer evaluation period. Atraumatic injuries were defined as being ‘closed’, ‘not open’ and ‘no trauma detected plus indoor cat’. Therefore, atraumatic injury was assumed, as the cats were not under observation when the CCT injuries were sustained. However, a traumatic event in the absence of an accident scenario without any access to outdoors seems unlikely.

## Conclusions

The results of this study partially support our initial hypothesis. Cats with atraumatic injuries had a higher mean BW than cats with traumatic injuries, but the difference was not statistically significant. We found that older cats were more likely to suffer atraumatic AT injuries.

The most common injury type according to the Meutstege classification system was type IIc, but anatomical differences between cats and dogs must be considered. Future studies should focus on the role of the SMT in the mechanism of AT injuries.

## Supplemental Material

Table 1 Synopsis of patient data, injury specifications, therapy and outcome in 66 cats

## References

[1] Mughannam A Reinke JR . Avulsion of the gastrocnemius tendon in three cats. J Am Anim Hosp Assoc 1994; 30: 550–556.

[2] Voss K Langley-Hobbs SJ Montavon PM . Tarsal joint. In: Montavon PM Voss K Langley-Hobbs SJ (eds). Feline orthopedic surgery and musculoskeletal disease. Philadelphia, PA: Saunders/Elsevier; 2009, pp 507–525.

[3] Cervi M Brebner N Liptak J . Short- and long-term outcomes of primary Achilles tendon repair in cats: 21 cases. Vet Comp Orthop Traumatol 2010; 23: 348–353. 20740261 10.3415/VCOT-09-10-0109

[4] Meutstege FJ . The classification of canine Achilles’ tendon lesions. Vet Comp Orthop Traumatol 1993; 6: 53–55.

[5] Reinke JD Mughannam AJ Owens JM . Avulsion of the gastrocnemius tendon in 11 dogs. J Am Anim Hosp Assoc 1993; 29; 410–418.

[6] Wong HK Bush AM Hoffmann DE . Flexor digitorum lateralis tendon transposition for the repair of bilateral calcaneal tendon rupture in a cat with severe thermal injury. Vet Comp Orthop Traumatol 2016; 29: 89–93. 26640837 10.3415/VCOT-15-01-0004

[7] Sangion F Cinti F Pisani G . Common calcaneal tenorrhaphy revision using a central gastrocnemius turnover aponeurotic flap technique in a cat. Vet Comp Orthop Traumatol 2018; 31: 67–70. 29325195 10.3415/VCOT-17-04-0060

[8] King M Jerram R . Achilles tendon rupture in dogs. Compend Contin Educ Pract Vet 2003; 25: 613–620.

[9] Nielsen C Pluhar GE . Outcome following surgical repair of Achilles tendon rupture and comparison between postoperative tibiotarsal immobilization methods in dogs: 28 cases (1997–2004). Vet Comp Orthop Traumatol 2006; 19: 246–249. 17143398

[10] Corr SA Draffan D Kulendra E , et al. Retrospective study of Achilles mechanism disruption in 45 dogs. Vet Rec 2010; 167: 407–411. 20834000 10.1136/vr.c4190

[11] Morton MA Thomson DG Rayward RM , et al. Repair of chronic rupture of the insertion of the gastrocnemius tendon in the dog using a polyethylene terephthalate implant. Vet Comp Orthop Traumatol 2015; 28: 282–287. 25804524 10.3415/VCOT-14-08-0133

[12] Morton MA Whitelock RG Innes JF . Mechanical testing of a synthetic canine gastrocnemius tendon implant. Vet Surg 2015; 44: 596–602. 26114897 10.1111/j.1532-950X.2015.12329.x

[13] Zellner EM Hale MJ Kraus KH . Application of tendon plating to manage failed calcaneal tendon repairs in a dog. Vet Surg 2018; 47: 439–444. 29393973 10.1111/vsu.12775

[14] Van Hagen MAE Ducro BJ van den Broek J , et al. Incidence, risk factors, and heritability estimates of hind limb lameness caused by hip dysplasia in a birth cohort of Boxers. Am J Vet Res 2005; 66: 307–312. 15757132 10.2460/ajvr.2005.66.307

[15] Brunnberg L Waibl H Lehmann J . Lahmheit beim Hund. Kleinmachnow: Procane Claudo Brunnberg; 2014.

[16] Kramer M Gerwing M Michele U , et al. Ultrasonographic examination of injuries to the Achilles tendon in dogs and cats. J Small Anim Pract 2001; 42: 531–535. 11721980 10.1111/j.1748-5827.2001.tb06022.x

[17] So V Pollard H . Management of Achilles tendon disorders. A case review. Australas Chiropr Osteopathy 1997; 6: 58–62. 17987151 PMC2050628

[18] Vosseller JT Ellis SJ Levine DS , et al. Achilles tendon rupture in women. Foot Ankle Int 2013; 34: 49–53. 23386761 10.1177/1071100712460223

[19] Pardes AM Freedman BR Fryhofer GW , et al. Males have inferior Achilles tendon material properties compared to females in a rodent model. Ann Biomed Eng 2016; 44: 2901–2910. 27150673 10.1007/s10439-016-1635-1 PMC5045781

[20] De la Riva GT Hart BL Farver TB , et al. Neutering dogs: effects on joint disorders and cancers in Golden Retrievers. PLoS One 2013; 8. DOI: 10.1371/journal.pone.0055937. 10.1371/journal.pone.0055937 PMC3572183 23418479

[21] Kiefel C Kutzler MA . Luteinizing hormone receptor expression in canine anterior cruciate and femoral head ligaments. Proceedings of the International Symposium on Canine and Feline Reproduction; 2016 Jun 22–25; Paris, France.

[22] Leppilahti J Orava S . Total Achilles tendon rupture. A review. Sports Med 1998; 25: 79–100. 9519398 10.2165/00007256-199825020-00002

[23] Maffulli N . Current concepts in the management of subcutaneous tears of the Achilles tendon. Bull Hosp Jt Dis 1998; 57: 152–158. 9809181

[24] Alfredson H Lorentzon R . Chronic Achilles tendinosis: recommendations for treatment and prevention. Sports Med 2000; 29: 135–146. 10701715 10.2165/00007256-200029020-00005

[25] Kannus P Józsa L . Histopathological changes preceding spontaneous rupture of a tendon. A controlled study of 891 patients. J Bone Joint Surg Am 1991; 73: 1507–1525. 1748700

[26] Józsa L Reffy A Kannus P , et al. Pathological alterations in human tendons. Arch Orthop Trauma Surg 1990; 110: 15–21. 2288799 10.1007/BF00431359

[27] Järvinen TA Kannus P Paavola M , et al. Achilles tendon injuries. Curr Opin Rheumatol 2001; 13: 150–155. 11224740 10.1097/00002281-200103000-00009

[28] Christensen J Alfredson H Andersson G . Protease-activated receptors in the Achilles tendon – a potential explanation for the excessive pain signalling in tendinopathy. Mol Pain 2015; 11: 13. DOI: 10.1186/s12990-015-0007-4. 25880199 10.1186/s12990-015-0007-4 PMC4369088

